# Autism spectrum disorder in older adults: The case study of a 65-year-old man

**DOI:** 10.1192/j.eurpsy.2022.1666

**Published:** 2022-09-01

**Authors:** J. Menstell, U. Altunoz, M. Ziegenbein

**Affiliations:** 1 Wahrendorff Clinic, Centre Of Transcultural Psychiatry & Psychotherapy, Sehnde, Germany; 2 Hannover Medical School, Research Group For Social And Transcultural Psychiatry & Psychotherapy, Hannover, Germany; 3 Wahrendorff Clinic, Department Of Psychiatry & Psychotherapy, Sehnde, Germany

**Keywords:** differential diagnosis, old age psychiatry, Autismus Spectrum Disorder

## Abstract

**Introduction:**

Autism spectrum disorder (ASD) is a neurodevelopmental disorder characterized with ritualized behavior, difficulties in communication/ social interaction, restricted interests, and sensitivity to external stimuli. The ASD has gained attention in recent years, however it’s still difficult in geropsychiatric setting to identify high-functioning ASD, especially when patients’ coping mechanisms are successful. Not to determine high-functioning ASD structure in older age can lead to wrong diagnosis and inappropriate treatment trials.

**Objectives:**

The aim of this presentation is to emphasize the importance of the evaluation of ASD-structure in old-age-psychiatry through the case study of a 65-years-old man.

**Methods:**

One case report from the inpatient unit of a psychiatric clinic in Lower Saxony, Germany will be presented.

**Results:**

Case: The patient was referred to our acute-psychiatric-ward due to delusional thoughts, depressive symptoms and lorazepam dependency. Delusional disorder was diagnosed in the outpatient-setting since he had interpreted some external stimuli in an eccentric way. During the therapeutic process, some features of high-functioning ASD such as social difficulties, dislike of change and repetitive/restrictive habits were prominent. Developmental history of the patient and the Autism-Spectrum-Quotient-50 also supported the clinical diagnosis of the ASD. Delusional disorder was excluded, and the therapy organized according to the structure characteristics of the high-functioning ASD which yielded to significant amelioration of depressive symptoms and increased perceived life quality of the patient.

**Conclusions:**

Although coping mechanisms of the patients can be successful, identifying high-functioning ASD-structure even in an old-age can be quite helpful in diagnostic/therapeutic processes. An elaborate discussion of the subject through contemporary literature will be presented.

**Disclosure:**

No significant relationships.

